# Land use influences the faecal glucocorticoid metabolites of multiple species across trophic levels

**DOI:** 10.1093/conphys/coae091

**Published:** 2025-01-20

**Authors:** Antje Chiu-Werner, Kerry V Fanson, Elissa Cameron, Menna Jones

**Affiliations:** College of Science and Engineering, School of Natural Sciences, University of Tasmania, Private Bag 55, Hobart, TAS 7001, Australia; Department of Animal, Plant and Soil Sciences, School of Agriculture, Biomedicine and Environment, La Trobe University, Biology Drive, Bundoora, VIC 3086, Australia; School of Biological Sciences, University of Canterbury, Private Bag 4800, Christchurch 8041, New Zealand; College of Science and Engineering, School of Natural Sciences, University of Tasmania, Private Bag 55, Hobart, TAS 7001, Australia

**Keywords:** Adrenal, cortisol, faeces, FGM, marsupials, stress, wildlife

## Abstract

Human landscape modification is amongst the greatest drivers of biodiversity loss. Measuring faecal glucocorticoid metabolites (FGM) in wildlife is of great value to measure the impact of human activities on local biodiversity because FGM offer a non-invasive way of measuring an animal’s response to changes in its environment in the form of adrenocortical activity. Here, we measure the concentration of FGM in three native Australian mammal species belonging to different trophic levels: the Tasmanian devil (*Sarcophilus harrisii*) and the spotted-tailed quoll (*Dasyurus maculatus*), both carnivores, and an omnivore that is primarily an arboreal folivore, the brushtail possum (*Trichosurus vulpecula*), and compare the FGM concentrations across three major land uses: agricultural, plantation and National Parks. We find that land use influences the FGM concentration in all three species and that general patterns emerge in FGM concentrations across multiple species and trophic levels in relation to land use. Specifically, plantation landscapes are associated with the lowest median and range of variation of FGM concentration in all species with several plausible explanations depending on the species. Our results suggest that measuring FGM in multiple species can offer a time- and cost-efficient snapshot of how different animals experience the same environment, potentially simplifying FGM interpretation. This study is the first to apply a community approach to understand how multiple species of different trophic levels respond collectively, and separately, to different land use types.

## Graphical Abstract

**Graphical Abstract:**
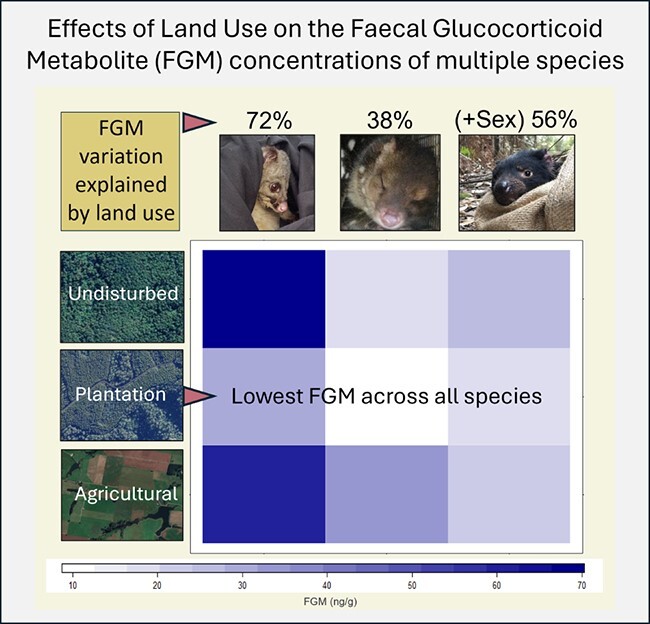

## Introduction

Biodiversity underpins the survival of humanity, yet its ongoing decline is driven primarily by human overexploitation and land use (Jaureguiberry *et al.,* 2022). Reducing the impacts of land use on biodiversity requires understanding how species cope with environmental change. Patterns of species occurrence and abundance in natural ecosystems (i.e. where human intervention is minimal and conservation efforts aim to maintain the ecosystem’s natural state) and managed ecosystems (where human activities play a significant role in shaping the environment) result from a combination of the influence of environmental change on ecological processes such as species interactions (e.g. Harpole and Tilman, 2007; Van der Putten *et al.,* 2010), intrinsic life history (the species-specific patterns and timing of reproduction, growth and longevity) and physiological traits (e.g. homeostasis, behaviour, health, fitness; Romero and Wikelski, 2010; Wingfield, 2013; Madliger and Love, 2015). Measuring physiological traits is useful because it can provide insights into how wild animals, rather than humans, perceive environmental change (Madliger and Love, 2015; Jones and Davidson, 2016).

Glucocorticoids (GCs), such as cortisol and corticosterone, are a particularly useful physiological trait to monitor because they regulate multiple aspects of physiology, including energy regulation, circadian rhythm, reproduction and stress response (Smith and Vale, 2006; Cain and Cidlowski, 2017). They are sensitive to external stimuli and can be monitored non-invasively. Although they are often thought of as stress hormones, GCs respond to a wide range of stimuli (e.g. circadian rhythms, seasons, perceived threats, injuries, disease, etc.) and are essential for life because they regulate carbohydrate metabolism, immune responses, life-history events (e.g. growth, migration, reproduction, metamorphosis, etc), amongst other processes (Oakley and Cidlowski, 2013; MacDougall-Shackleton *et al.,* 2019). As a response to a stimulus, the adrenal cortex releases GCs into the bloodstream, where they exert modulating and preparative actions (Sapolsky *et al.,* 2000) that result in behavioural and physiological coping mechanisms (Romero, 2004). Subsequently, GCs are metabolized in the liver and by microbiota during their transit through the intestines and eventually excreted in urine and faeces (Taylor, 1971; Palme *et al.,* 2005; Gérard, 2020). The concentration of GC metabolites obtained in faeces (FGM–faecal glucocorticoid metabolites) can therefore be used to obtain an integrated measurement of adrenocortical activity (Palme, 2019). Because of the complexity in the production and use of GCs, the proportions of GCs excreted in faeces and urine differs amongst species, sexes and individuals and thus requires validation for each species and sex (Touma *et al.,* 2003; Palme *et al.,* 2005).

Land use has generally been found to influence the FGM concentrations of wildlife species (e.g. Spercoski *et al.,* 2012; Tecot, 2013; Pokharel *et al.,* 2019; Brunton *et al.,* 2020; Boyle *et al.,* 2021). However, the response to changes in land use varies amongst species. Whilst some studies establish a positive correlation between adrenal activity and human disturbance (Spercoski *et al.,* 2012; Creel *et al.,* 2013a; Bókony *et al.,* 2021), others show the opposite response (Tecot, 2013; Pokharel *et al.,* 2019) or a combination of responses (e.g. Brunton *et al.,* 2020,), and yet other species show no differences in the FGM concentrations of populations inhabiting different land uses (e.g. Lèche *et al.,* 2014; Ramahlo *et al.,* 2019). Interpreting FGM in wildlife living in their natural environment poses several challenges because FGM represent the end product of hormones (GCs) that are released in response to multiple external stimuli, and the metabolism of which can be affected by individual differences such as sex, age, diet, metabolic rate and composition of gut bacteria (Goymann, 2012).

In this study, we evaluate a multi-species or community approach to using FGM to measure how different species are responding to the same environmental challenges within a specific land use type at the same time, to better understand the true impacts of land use on wildlife. We focus on three sympatric native marsupial species in Tasmania, Australia, representing two different trophic levels. Specifically, we measure the concentration of FGMs in two hyper-carnivores—the Tasmanian devil (*Sarcophilus harrisii*) and the spotted-tailed quoll (*Dasyurus maculatus*)—as well as an arboreal omnivore that primarily consumes plant leaves: the brushtail possum (*Trichosurus vulpecula*), and compare their FGM concentrations amongst the three major land use types in Tasmania: undisturbed forest (within National Parks), forestry plantations (eucalypt or pine monocultures) and agricultural land (vegetation remnants surrounded by agricultural or farming land). By including species with different ecological roles (i.e. trophic levels), we expect FGMs to capture the effects of a shared landscape on environmental factors such as prey availability (e.g. Chiu-Werner and Jones, 2023; Lewis *et al.,* 2023) and predation risk, which affect predators and prey in different ways (Creel *et al.,* 2013b). We aim to answer the following questions: 1) Does land use influence the FGM concentrations of the wildlife living in these landscapes, and 2) are there consistent patterns in the response to land uses across multiple species and trophic groups when measured?

## Materials and Methods

### Species, study sites and field methods

We measured FGM concentrations in three widespread native mammals at two trophic levels, hyper-carnivores and omnivores. The carnivores were the Tasmanian devil, a threatened non-territorial top predator, and the spotted-tailed quoll, a threatened mesopredator that is partially arboreal female territoriality (Baker and Gynther, 2003). The omnivore was the common brushtail possum, a socially monogamous and territorial species, which is primarily an arboreal folivore that also forages on the ground. All three species are nocturnal and were captured by setting traps overnight, baited with either meat for the carnivores or peanut butter and rolled oats for the omnivore.

**Figure 1:**
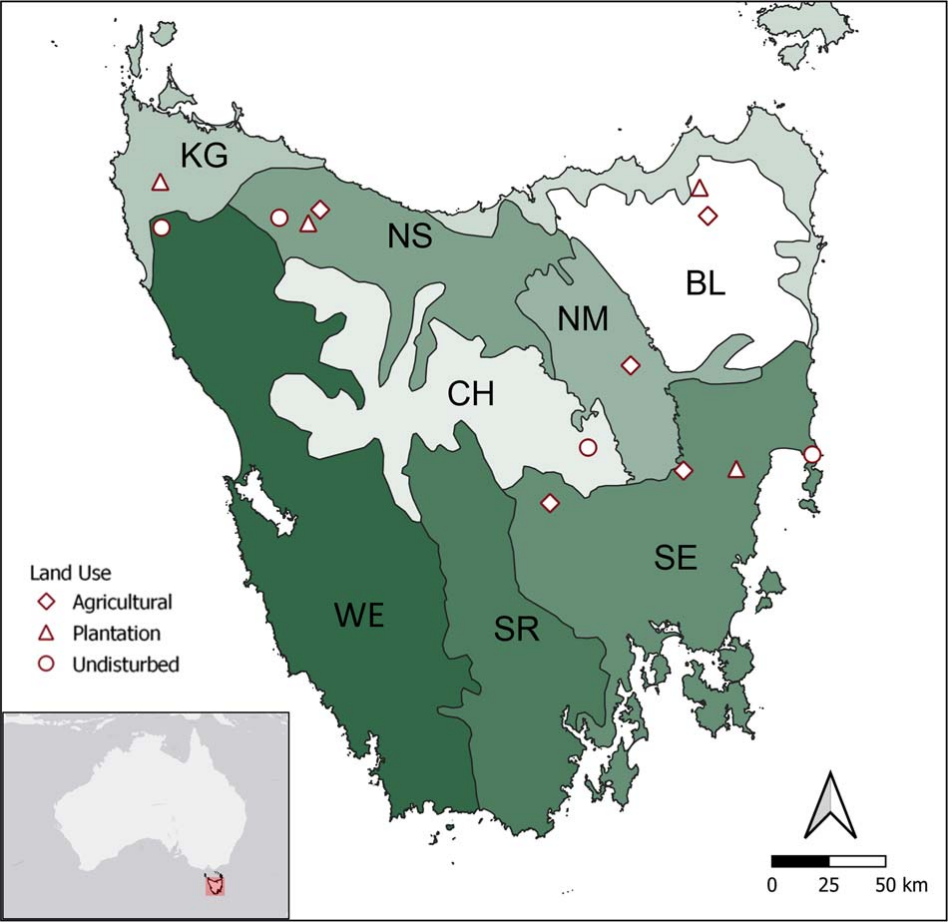
Location of sampling sites across bioregions in Tasmania. KG = King, NS = Northern Slopes, BL = Ben Lomond, NM = Northern Midlands, CH = Central Highlands, SE = South East, SR = Southern Ranges, WE = West (Peters and Thackway, 1998). Reproduced with permission from Chiu-Werner and Jones (2023).

Study sites were selected following Chiu-Werner and Jones (2023) to account for land use and geographical variation. Briefly, we sampled 13 study sites, each ~25 km^2^ in size, within each of the three major land uses in Tasmania across six different bioregions (Peters and Thackway, 1998), where these land use types were present. Sampling the bioregions accounted for geographic and climatic variation across the island (Fig. 1). Land use was defined as ‘Undisturbed forests’ (hereafter ‘Undisturbed’) if located within a National Park with at least 80% of the area covered by undisturbed native vegetation (*n* = 4). Undisturbed landscapes allow for public visitation with minor supervision, and traffic can take the form of bushwalkers around established paths, and cyclists, motorbikes and cars on unsealed roads. Visitor numbers are highly skewed towards the austral summer months, being much less during winter when sample collection took place. ‘Forestry plantations’ (hereafter ‘Plantation’) were areas with at least 60% cover of mature (>15 years) pine or eucalypt plantations (*n* = 4). Edges in the form of roads and ecotones between plantation and native remnants abound in these landscapes. Traffic of cars and trucks is limited and restricted to roads, and usually related to forestry patrol/supervision and research activities. No sampling occurred in plantation landscapes where harvesting or thinning was taking place. Finally, ‘Agricultural’ landscapes consisted of vegetation remnants (native or plantations) within a landscape that had at least 80% of its area covered by agricultural or farming land (*n* = 5).

Field trapping was undertaken once at each site during the austral winter, between the end of April and the end of August 2021, when temperatures were consistently low, to minimize seasonal variation in temperature that may influence both GC production and degradation of FGM in faeces prior to collection. At each site we deployed 10 small-to-medium and 20 medium wire cage traps, and 10 dedicated carnivore pipe traps for five consecutive nights. Trap types and baits targeted different functional groups: meat for carnivores and peanut butter/rolled oats for omnivores. Traps were placed in sheltered locations and covered with hessian bags to provide refuge and checked from dawn every morning. By checking traps at dawn, we ensured the collection of faeces occurred within a 12-h window after excretion to reduce FGM degradation (Millspaugh and Washburn, 2004) and when temperatures were at their minimum. Average overnight temperatures on sampling sites varied between 2.8°C and 5.6°C (BOM, 2021) during our trapping period. We mixed up the geographic pattern of trapping to ensure that we did not establish seasonal patterns in the results.

We collected faeces from the floor of each trap, placed them in zip-lock bags, and kept them in a cooler box on ice until arrival at the accommodation where we proceeded with steroid extraction (details in section below) on the same day.

### Biological validation

Biological validation for FGMs of Tasmanian devils and brushtail possums has been previously conducted and was not repeated here (Keeley *et al.,* 2012; Fanson *et al.,* 2017; Cope *et al.,* 2022).

For spotted-tailed quolls, we used capture/recapture of the same individuals within the same 10-day field trapping trip to compare two assays. We used the faecal sample collected from the trap during the first capture as a baseline for FMG concentrations, and faecal samples collected during subsequent recaptures to measure an expected increase in adrenal activity resulting from the time spent in the trap and handling during the first capture. We expected FGM concentrations to decrease ~4 days after the first capture (Fanson *et al.,* 2017).

In November 2020, 6 months prior to the main field trapping programme, we joined a University of Tasmania carnivore trapping trip being done to study devil facial tumour disease (DFTD), where spotted-tailed quolls are a frequent bycatch. To record the time of the night when each animal was trapped, we positioned a camera trap <5 m from and facing the entrance to the trap. For initial captures, processing takes an average of 7 min. This includes inserting a microchip, collecting ear biopsies and a whisker sample, recording sex (and reproductive condition from the pouch if female), weight, head and tail width, and the length of over-eruption in the canine teeth (for ageing) (Jones, 2023). For trip recaptures, animals are released immediately following identification (with an average processing time of 20 s), whilst recaptures from previous trips are measured and checked for general body condition (pouch is checked if female). We note that if an individual hyperventilated whilst being handled (which is interpreted as extreme distress), it is released immediately without further examination. This occurred with one male, for which there is only the initial sample as it was not caught again. We captured a total of nine individuals (seven males and two females). We obtained five faecal samples for one male (Days 1, 4, 6, 8 and 10), and two faecal samples for each of two females (Days 1 and 3, and Days 1 and 2, respectively), where Day 1 represents the collection of the baseline faecal sample. Following Fanson *et al.* (2017), we defined an assay as successful if their mean Z-score (i.e. strength of the response to first capture) was ≥2, and if the assay detected a peak in at least 50% of the individuals.

### Extraction and analysis of the faecal glucocorticoid metabolites

Steroid metabolites were extracted by adding 5 ml of 80% methanol to 0.5 g (± 0.01 g) of homogenized, wet faecal material in a 10-ml polypropylene tube. If the sample was <0.5 g we reduced the amount of alcohol accordingly to maintain the 1:10 sample:methanol ratio. The samples were then shaken vigorously by hand until the faecal material broke apart (~8 min) and allowed to settle overnight before transferring 1 ml of the supernatant to a 1.5-ml polypropylene screw-top vial (Shutt *et al.,* 2012). Extracts were stored at room temperature (~15°C) until analysis (Terio *et al.,* 2002; Palme *et al.,* 2013).

For Tasmanian devils, FGM were quantified using a corticosterone enzyme immunoassay. The antibody (lab product code Cs6) and corresponding horseradish peroxidase conjugate were developed at the University of California, Davis (first described by Watson *et al.,* 2013). This assay has been previously validated for monitoring FGM in Tasmanian devils (Keeley *et al.,* 2012; Fanson *et al.,* 2017).

For spotted-tailed quolls, we compared the performance of two different assays that have been validated in closely related dasyurid species: (1) the Cs6 assay described above for Tasmanian devils, and (2) a broad-spectrum antibody that had been validated for western quolls (Jensen *et al.,* 2019); the antibody (lab product code 37e) and corresponding biotin-labelled hormone were developed at the University of Veterinary Medicine, Vienna (first described by Touma *et al.,* 2003).

For brushtail possums, FGM were analysed using a group-specific antibody raised against 11-oxoetiocholanolone. The antibody (lab product code 72-alt) and corresponding biotin-labelled hormone were developed at the University of Veterinary Medicine, Vienna (first described by Palme and Mostl, 1997). This assay has been previously validated for brushtail possums (Cope *et al.,* 2022).

Assay procedures were followed according to those previously described (Palme and Mostl, 1997; Keeley *et al.,* 2012; Fanson *et al.,* 2017; Jensen *et al.,* 2019). Briefly, 96-well micro-titer plates were coated with goat anti-rabbit IgG (Arbor Assays A009). Plates were washed and loaded with 50 μl of standard, control or diluted sample extract, followed by 50 μl of enzyme-labelled hormone and 50 μl of antibody. For the Cs6 assay, plates were incubated for 2 h and washed before adding 150 μl ABTS substrate solution. Optical density was measured at 405 nm (620-nm reference filter). For the 37e assay, plates were incubated overnight at 4°C and washed before adding 250 μl streptavidin–peroxidase solution. After 45 min, plates were washed again and 250 μl of TMB substrate solution was added. The reaction was stopped with 50 μl H_2_SO_4_ and optical density was measured at 450 nm (620 reference filter). Assays were biochemically validated by demonstrating parallelism between a serially diluted extract pool and the standard curve. To monitor precision and reproducibility, low (~70% binding)- and high (~30% binding)-quality control samples were run on each plate. The intra-assay coefficient of variation (CV) was <15% for all assays. The average inter-assay CV for Tasmanian devils was 2.6% (*n* = 2 plates), for brushtail possums 10.3% (*n* = 2 plates), whilst spotted-tailed quoll samples were run on a single plate. All samples were analysed on the same day to minimize variation. All FGM concentrations are expressed as nanogramme per gramme wet faeces.

### Data analysis

We used linear models with a Gaussian data family to first run a general model that included all assessed species as a fixed factor:

FGMconc ~ species + landUse + bioregion + sex + landUse^*^species.

We then assessed the extent to which land use, sex and bioregion explained FGM concentrations in each species with the following model:

Species_(FGMconc)_ ~ landUse + sex + bioregion.

All individuals of brushtail possums and spotted-tailed quolls were mature adults, whilst only four devils in our sample were aged >1 year. Florent *et al.* (2023) did not find age to influence FGM concentrations in devils, hence it was not included as a factor in the model. The over-representation of young devils in the population is attributed to the spread of DFTD (McCallum *et al.,* 2007; Lazenby *et al.,* 2018). At this stage devils are still considered young but are sexually mature and fully independent.

Linear models were fit using the R package *lme4* (Bates *et al.,* 2015). We log-transformed the FGM concentrations to achieve a normal distribution for the linear models and run diagnostics on the residuals. We applied a second-order Akaike’s information criterion for small sample sizes (AICc; Burnham and Anderson, 2002) to rank models using the *AICcmodavg* R package (Mazerolle, 2020) and selected the most parsimonious model for all and each species. We detected an outlier in the devil dataset (a female with FGM concentration 99.5 ng/g; nearly two times higher than the next value). We decided to remove this outlier despite obtaining the same resulting candidate models when including it in the analysis.

### Ethical declarations

The study was undertaken with approval of the University of Tasmania Animal Ethics Committee (permit A0018012); the Department of Primary Industries, Parks, Water and the Environment (permits TFA20079, TFA21035); Sustainable Timber Tasmania (permit FAA1829); and Reliance Forest Fibre (permit RFF052).

## Results

### Biological validation

For spotted-tailed quolls, assay *Cs6* detected a greater and more consistent increase in FGM at recapture compared to assay *37e*. *Cs6* measured an increase in FGM concentration in all three of the recaptured animals compared to the initial capture (Z-score = 34.8, 42.8 and 29.6; equivalent to increases of 1.5-fold, 2.1-fold and 3.2-fold, respectively). Assay *37e* only detected an increase at recapture for both females. This increase was smaller than *Cs6* (Z-score = 8.2 and 1.7; equivalent to 1.2-fold and 2.1-fold increases, respectively). For the male, FGM concentrations effectively decreased at all subsequent recaptures by 0.62-fold when measured with assay *Cs6*, whilst remaining constant when measured with assay *37e*. Although both assays detected a peak in at least 50% of the individuals, only assay *Cs6* had an average Z-score ≥5 (good signal-to-noise ratio; Fanson *et al.,* 2017). Consequently, we used *Cs6* to quantify FGM in spotted-tailed quoll samples. Assays were biochemically validated by demonstrating that the displacement curve for serial dilutions of faecal extracts was parallel to the standard curve. The relative variation of the slope of the trend lines was <5%.

### Field FGM results

**Figure 2:**
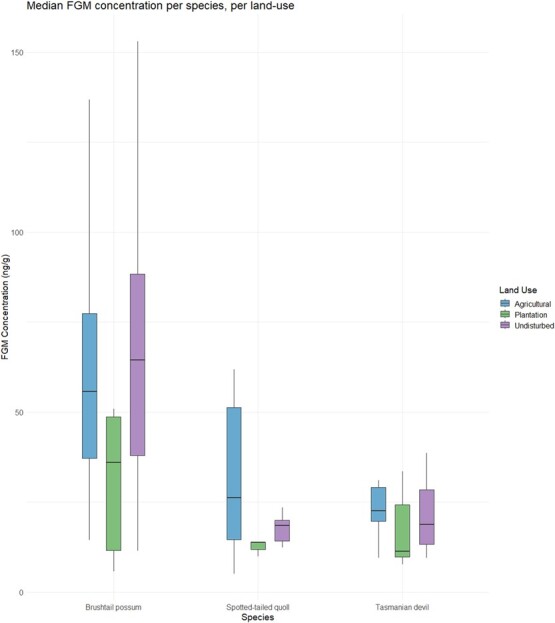
Boxplot showing median faecal glucocorticoid metabolites (FGM) concentration (nanogramme per gramme) across three species under different land use categories (Agriculture, Plantation and Undisturbed), with the lower and upper edges representing the 25th and 75th percentiles, and the whiskers indicating the variability in FGM concentrations across land use types.

We discovered two general patterns in our measurements of FGM. First, land use was an important parameter explaining the variation in FGM concentrations in all species; and second, animals inhabiting plantations all showed the lowest median in FGM concentrations whilst still responding differently to each land use (Fig. 2). Finally, we found species-specific differences in the FGM concentrations between sexes in the carnivores and amongst bioregions in possums (Table 2).

We obtained samples of 43 individual Tasmanian devils (Table 1), 18 spotted-tailed quolls and 40 brushtail possums. The median and upper and lower quartiles of FGM concentrations for each species and landscape are visualized in Fig. 2, with the values for median and range in Supplementary Table 1.

**Table 1:** Distribution of samples across land uses, bioregion and sexes

Land use	Species	Bioregion	Females (*n*)	Males (*n*)
Undisturbed	Tasmanian devil	Central Highlands	2	
		King	3	3
		Northern Slopes	5	6
		South East	3	2
	Spotted-tailed quoll	Northern Slopes	2	3
		South East		1
	Brushtail possum	Central Highlands	2	5
		Northern Slopes	1	
		South East		4
Plantation	Tasmanian devil	Ben Lomond	1	1
		King	3	2
		Northern Slopes	2	1
		South East		1
	Spotted-tailed quoll	Ben Lomond	1	
		Northern Slopes	1	
		South East		1
	Brushtail possum	Ben Lomond		2
		Northern Slopes	2	
		South East	1	
Agricultural	Tasmanian devil	Central Highlands	1	2
		Northern Slopes	2	3
	Spotted-tailed quoll	Ben Lomond		1
		Central Highlands	1	2
		Northern Midlands	1	3
		Northern Slopes		1
	Brushtail possum	Ben Lomond	6	1
		Central Highlands	4	2
		Northern Midlands	5	2
		Northern Slopes	1	
		South East	2	

The most parsimonious model resulting from the general model was an additive model that included ‘species’ and ‘land use’ and explained 95% of the FGM concentrations (Table 2a). The second model included the additive values of the variables ‘species’ and ‘land use’ and the interaction between both variables. The second model explained just 3% of the FGM concentrations. Quolls and devils both had lower FGM than possums, by 0.85 ng/g and 0.8 ng/g, respectively, and FGM values for all species in plantations were 0.5 ng/g lower than in both undisturbed and agricultural landscapes.

**Table 2:** Most parsimonious model results for the general model (2a) and each species models (2b), where Sp = Species, LU = Land Use, AICc = AIC value of the model corrected for small sample sizes (Burnham and Anderson, 2002), ΔAICc = difference between the AIC of the best model compared to the current model being compared, and AICc weight = proportion of the total predictive power that can be found in the model. The intercept for ‘Species’ is brushtail possum, for ‘Sex’ is female and for ‘Land Use’ is Undisturbed. Bioregions: CH = Central Highlands, KG = King, NM = Northern Midlands, NS = Northern Slopes, SE = South East. Parameters where the standard error is small relative to the effect size are highlighted in bold

2a.												
					**Parameter estimates ±** s**tandard error**							
					**Species**				**Land use**			
**Model**	**AICc**	**ΔAICc**	**AICc weight**	**Intercept**	**Spotted-tailed quoll**	**Tasmanian devil**		**Plantation**		**Agricultural**		
Sp + LU	185.85	0.00	0.95	**3.86 ± 0.13**	**−0.85 ± 0.17**	**−0.80 ± 0.14**		**−0.50 ± 0.16**		0.10 ± 0.14		
2b												
**Model**	**AICc**	**ΔAICc**	**AICc weight**	**Parameter estimates ±** s**tandard error**								
				**Intercept**	**Sex (M)**	**Land use**		**Bioregion**				
						**Plantation**	**Agricultural**	**CH**	**KG**	**NM**	**NS**	**SE**
**Tasmanian devil**												
Sex + LU	65.43	0.00	0.35	**3.20 ± 0.12**	**0.31 ± 0.15**	**−0.44 ± 0.19**	0.03 ± 0.22					
Sex	66.31	0.88	0.23	**3.10 ± 0.11**	−0.29 ± 0.16							
Sex + Bioregion	67.16	1.73	0.15	**3.29 ± 0.34**	−0.28 ± 0.15			−0.00 ± 0.40	−0.58 ± 0.37		−0.14 ± 0.35	0.03 ± 0.39
**Spotted-tailed quoll**												
Sex	40.54	0.00	0.62	2.75 ± 0.26	0.35 ± 0.32							
LU	41.51	0.97	0.38	2.85 ± 0.25		−0.33 ± 0.44	0.38 ± 0.33					
**Brushtail possum**												
LU	87.12	0.00	0.72	3.98 ± 0.19		**−0.86 ± 0.36**	−0.03 ± 0.24					
Bioregion	89.67	2.55	0.20	3.66 ± 0.22				**0.54 ± 0.29**		0.21 ± 0.33	−0.57 ± 0.40	0.26 ± 0.33
Sex	91.62	4.51	0.08	3.86 ± 0.15	0.003 ± 0.23							

In Tasmanian devils, the final candidate model set describing FGMs included three models with close AIC values, within 1.73 of each other (Table 2b). The top model contained the additive values of the variables sex and land use, and explained 35% of the FGM concentrations. The second model was nested within the top model, including only the variable sex, and explained 23% of the variation in FGM concentrations. The third model carried only 15% of the weight and included the additive values of the variables sex and bioregion. Females had 0.31 ng/g higher FGM concentrations than males with their parameter estimates showing low variation. Devils inhabiting plantation land uses had 0.44 ng/g lower FGM concentrations than those inhabiting undisturbed landscapes. The FGM estimates for agricultural landscapes were similar to those in undisturbed landscapes but these estimates were highly variable making comparisons less reliable. The inclusion of bioregion as a parameter in the candidate model set means bioregion has some influence but this is very weak, given the small AIC weight of the third model and the large variance of the parameter estimates.

For spotted-tailed quolls, there were two models in the final set that described differences in FGM concentrations. The top model contained only sex and carried 62% of the AIC model weight. The second model, containing only land use, was separated from the first by just 0.97 AIC values and carried 38% of the model weight (Table 2b). There was a large amount of variation around all these parameter estimates so the results are equivocal, but males had generally 0.35 ng/g higher FGM concentrations than females, and plantation land uses resulted in the lowest FGM concentrations in spotted-tailed quolls, with 0.33 ng/g and 0.71 ng/g lower FGM concentrations than undisturbed and agricultural landscapes, respectively.

In brushtail possums, differences in FGMs were described by three univariate models comprising the final model set (Table 2b). The top model included land use with an AIC weight of 72%. The second model contained the differences amongst bioregions, carrying 20% of the AIC weight. Differences in FGM concentrations between the sexes were in the third model, but this model carried little weight (8%). The lowest FGM concentrations were found in individuals captured in plantations, 0.86 ng/g lower than found in undisturbed landscapes. Brushtail possums in agricultural land uses had similar FGM concentrations to those in undisturbed land uses, although the variation around the parameter estimate was high. Individuals inhabiting the Central Highlands bioregion had 0.54 ng/g higher FGM concentrations than those in other bioregions with a trend for lower FGM in the Northern Slopes, albeit with the higher variance meaning that this parameter had less influence.

## Discussion

Our results indicate that land use is the most important factor explaining FGM variation after accounting for species. Our results also represent the first known measures of FGM for each of these species inhabiting specific land uses making their interpretation manyfold. One such interpretation relates to the population densities of each species. However, information on population abundances for each of these species in different land uses in Tasmania is limited. For instance, Tasmanian devil populations are below carrying capacity across the entire island due to the impacts of DFTD (Cunningham *et al.,* 2021). Densities of spotted-tailed quolls could be similar in agricultural and intact areas in Tasmania, as found in mainland Australia (Henderson *et al.,* 2022), whilst densities of brushtail possums in Tasmania are generally variable (le Mar and McArthur, 2005). Given that devil populations are below carrying capacity and quolls are naturally rare, the interpretation from a population density will be applied primarily to possums and assume that our capture rates reflect species abundance in each landscape. Conversely, the trophic roles of all three species are better known, and previous studies have shown land use to influence the diet of these three species (Chiu-Werner and Jones, 2023; Lewis *et al.,* 2023,), and diet to impact FGM concentrations (Kalliokoski *et al.,* 2015). Thus, our discussion will primarily focus on the ecological role of each species.

At first, finding the lowest FGM levels in plantation landscapes in all assessed species appears counter-intuitive if we assume that lower FGM concentrations are equivalent to lower stress levels. Indeed, GCs are often equated with stress (MacDougall-Shackleton *et al.,* 2019,), and consequently animals with higher FGM are assumed to be more stressed. However, there is a growing body of literature highlighting that interpretation of GCs is not that simple. GCs are involved in many aspects of physiology, and predictable fluctuations are essential for survival (Fanson, 2019; Romero and Beattie, 2022). For example, circadian fluctuations in GCs can be similar in magnitude to acute stress responses, and dampened rhythms are associated with pathologies such as depression or post-traumatic stress disorder (Linklater *et al.,* 2010; Romero and Beattie, 2022). Low GC levels may also be a sign of adrenal fatigue, potentially indicating unhealthy individuals (Romero and Beattie, 2022). Hence, when making inferences about the fitness consequences of observed patterns of adrenal activity, we must not assume a simple relationship between GC level and stress.

Taking this into account, two opposing interpretations could explain the result that these three wildlife species present with the lowest FGM values in plantations. The first interpretation is that it is possible that plantation land uses provide a predictable environment for wildlife, at least when there are no forestry activities taking place. The plantation landscapes we sampled were mature (i.e. >15 years of age) with no current forestry management activities. In Tasmania, 90% of the hardwood (*Eucalyptus globulus* and *Eucalyptus nitens*) plantations are managed without thinning operations and clear-felling, which in any case typically happens at 10–15 years of age (Wood *et al.,* 2008). The intervals between these management operations are longer than the average lifespan of most of the native species inhabiting them (e.g. Tasmanian devils in a healthy population free of facial tumour disease typically live for up to 5–6 years, (Guiler, 1978; Jones *et al.,* 2008),), and the GC timeframe that the FGM measure represents is comparatively very short, ~4 days (Fanson *et al.,* 2017). Plantations provide large amounts of edge habitats along roads and boundaries with native vegetation remnants, which provide ideal hunting opportunities for carnivores (Andersen *et al.,* 2017). Vehicular and pedestrian traffic are minimal outside of forestry operations and are restricted to roads, which wildlife could perceive as regular events, which can be perceived as environmental predictability and to which they may habituate. Interpreting the FGM results of brushtail possums in plantations is more challenging as previous studies, which measured possum density in different habitats in a plantation landscape, infer that plantations in Tasmania represent low-quality environments for this species (le Mar and McArthur, 2005). This would be supported by the low number of possum captures in this landscape assuming our capture numbers reflect this species abundances in each landscape. In this case, from an individual possum perspective, low population densities in plantations could result in reduced intra-specific competition for food and shelter and reduced stress-induced adrenocortical activity (Creel *et al.,* 2013b).

A second, contrasting interpretation is that the low FGM levels found in all species in plantations may represent a suppression of normal adrenal activity and might indicate an issue with adrenal function in response to how animals are perceiving their environment (Romero and Beattie, 2022). For possums, as a prey species, plantations could represent environments with high predatory pressure because plantations provide easy hunting grounds in the form of linear features for devils and quolls (Andersen *et al.,* 2017; Scoleri *et al.,* 2023). Changes in the behaviour of prey will affect the behaviour of predators in what is called reciprocal phenotypic plasticity, e.g. (Kishida *et al.,* 2006) which could also represent stressful events for predators.

We acknowledge that in both scenarios only three spotted-tailed quolls were captured in plantation landscapes (Table 1). Remarkably, the variation in FGM concentration in this species was the lowest of the three species, despite the small sample size and that each individual quoll was captured in a different bioregion.

Our models suggest similar effects of both agricultural and undisturbed land uses on FGM concentrations for all three species. However, there is considerable variation in possums and quolls, reducing the confidence in the parameter estimates representing meaningful effects. In agricultural landscapes, physiological changes associated with loss of habitat could be offset by benefits such as increased foraging and hunting opportunities, food subsidies (e.g. roadkill, crops) and shelter (le Mar and McArthur, 2005; Harper *et al.,* 2008; Andersen *et al.,* 2017). Such an interpretation would be in line with Pokharel *et al.* (2019), who found that benefits of increased food quality may reduce the human-induced stress response in wild Asian elephants (*Elephas maximus*). Like plantations, agricultural landscapes offer many linear features that are favoured by predators. This is reflected in a reduced dietary niche in devils (Lewis *et al.,* 2023), but it also shows the opportunistic behaviour of devils and quolls when food subsidies are present (Chiu-Werner and Jones, 2023). Ecological aspects (i.e. easy access to food) and dietary/alimentary processes (i.e. increased or reduced defecation) both may also influence the total FGM output in both agricultural and plantation land uses in both carnivore species. For possums, agricultural landscapes offer a variety of plants that are lower in plant secondary metabolites, which would be favoured (Scrivener *et al.,* 2004), but also opportunities for shelter (e.g. sheds, roofs, vegetation remnants) with potential influences on adrenal activity. In quolls, similar responses in agricultural and undisturbed land uses could also be attributed to potential similarities in population densities (Henderson *et al.,* 2022). Finally, the amount of stochastic human disturbance may be higher in agricultural than in plantation landscapes simply because people live in agricultural landscapes and there are more dogs, movement of livestock, machinery and potentially shooting of macropod wildlife. Plantation landscapes could simply be quieter.

In undisturbed landscapes, high variability in food availability and quality may impose physiological stressors on wildlife (Zweifel-Schielly *et al.,* 2009; Bertuzzo *et al.,* 2016; Courtemanch *et al.,* 2017) resulting in higher FGM concentrations and intra-specific variability thereof. This is because Protected Areas in Tasmania, like elsewhere in the world (Margules and Pressey, 2000), are often located in rugged areas with low soil fertility that are not productive or useful for forestry or agriculture. But higher variability in FGM could also reflect healthy fluctuations in adrenal activity, associated with these challenges, which could be suppressed in populations in plantations (Tecot, 2013).

Sex also exerted an influence on the FGM concentrations in all species. This is expected because of the involvement of sex steroids in the response of males and females to stress (Tilbrook *et al.,* 2000) and the differences in energetic and reproductive demands between males and females (Aloise King *et al.,* 2013). In all species however, the variation around the parameter estimate was large. The collection of samples during the austral winter may affect the species differently due to their mating periods and social organization. In winter, sexual competition in devils is at its lowest in the year but females are carrying and provisioning young in the pouch, which could explain that females have higher FGM concentrations than males. Agonistic behaviour in this non-territorial species is associated with the late summer and autumn mating period (Baker and Gynther, 2003). We hypothesise that if sampling had occurred during the devil mating season, sexual competition might have overridden other environmental factors such as land use (e.g. Eggermann *et al.,* 2013), resulting in lower variability of FGM concentrations. Spatial organization differs between sexes in spotted-tailed quolls, with females being territorial and male home ranges overlapping several female territories (Körtner *et al.,* 2004; Andersen *et al.,* 2017). Our sampling period overlapped their breeding peak in June and July and birthing period in July and August (Belcher, 2004), when males can fight for access to females (Jones *et al.,* 2001), which would explain male quolls having higher FGM concentrations than females albeit with high variation around the parameter estimate. Likewise, birthing in possums, which are solitary and territorial, occurs between April and the end of June, and joeys are carried in the pouch for up to 6 months (Hocking, 1981). Being off the mating season could explain the large variation around the parameter estimate of males (0.003 ± 0.23 ng/g) in relation to females in this species. In spotted-tailed quolls, an additional explanation to the importance of sex as a factor explaining FGM variation might be related to our sample distribution, which was heavily biased towards male individuals (Table 1).

Bioregion had weak influence on FGM concentrations and only in possums, with higher FGM concentrations only in the Central Highlands bioregion. This result may reflect an influence of agricultural landscapes, which are the only sites sampled in this bioregion. Whilst possum FGM did not respond to agricultural landscapes across Tasmania, the agricultural landscapes in the Central Highlands are highly fragmented and exposed, with constant wind and wind chill in a climate that is cold and wet for much of the year. This area is climatically the harshest bioregion in Tasmania, which could cause physiological stress for arboreal nocturnal animals that forage in these open, degraded and fragmented forests. This stress may be less for terrestrial predators, such as devils and quolls, that hunt along forest edges on the ground (Andersen *et al.,* 2017).

Our study highlights that anthropogenic land use influences animal physiology, though further studies are needed to understand how these differences relate to animal fitness. The physiological response of wildlife to human disturbances, including land use, is complex. Studies measuring FGM concentrations as an indicator of stress (e.g. Spercoski *et al.,* 2012; Tecot, 2013; Creel *et al.,* 2013a; Pokharel *et al.,* 2019; Brunton *et al.,* 2020; Bókony *et al.,* 2021) show that each response to a human disturbance is unique even within the same species. For example, Brunton *et al.* (2020) found two different populations of eastern grey kangaroos (*Macropus giganteus*) to show opposite responses to living in urban environments, with one population having higher FGM concentrations in urban than in non-urban areas, and another population at a different location showing the reverse. Considering the amount of intra-specific variability in FGM concentrations, it is surprising that few studies (e.g. two species, Creel *et al.,* 2002) have measured the physiological responses of multiple species within communities to human disturbances.

Our study results, indeed, raise a lot of questions because they are the first to measure FGM concentrations in multiple species of different trophic levels in the same and multiple environments, and because the interpretation of FGM concentrations in free-living animals is complex (Reeder and Kramer, 2005; Goymann, 2012; Romero and Beattie, 2022). However, our results also show patterns across species. These patterns are valuable for measuring and monitoring the impact of human activities on local biodiversity using FGM analysis and they should be further validated by comparison with other methods such as video recordings of behaviour (Fardell *et al.,* 2021). Measuring FGM concentrations of multiple species could be used in environmental impact assessments on terrestrial ecosystems to monitor the effects of human activities over time and assess if species habituate or show new adaptations to these environmental changes. For example, how do plantations influence FGM at different stages of establishment and ages of growth. The key advantage of monitoring human impacts on the environment through measuring FGM in multiple species is that faeces are abundant and do not require invasive methods for collecting. Faecal samples can be collected in a short field season to reduce environmental stochasticity and are easily assessed for age (e.g. Vynne *et al.*, 2011) to reduce the influence of environmental exposure (Lafferty *et al.,* 2019). Finally, if biological validation has been done for the species involved (Palme, 2019), our multi-species approach to FGM analysis is a time- and cost-efficient way to convey a snapshot of how different animals are perceiving the same environment.

## Supplementary Material

graphAbstract2_coae091

## Data Availability

*The data underlying this article are available in* figshare, at https://figshare.com/s/4ace4c84ecc0424d403d
