# Supplementary figures and images for: Land use influences the faecal glucocorticoid metabolites of multiple species across trophic levels
Source: Conserv Physiol. 2025 Jan 20;13(1):coae091. doi: 10.1093/conphys/coae091 (PMC11744367; doi:10.1093/conphys/coae091)

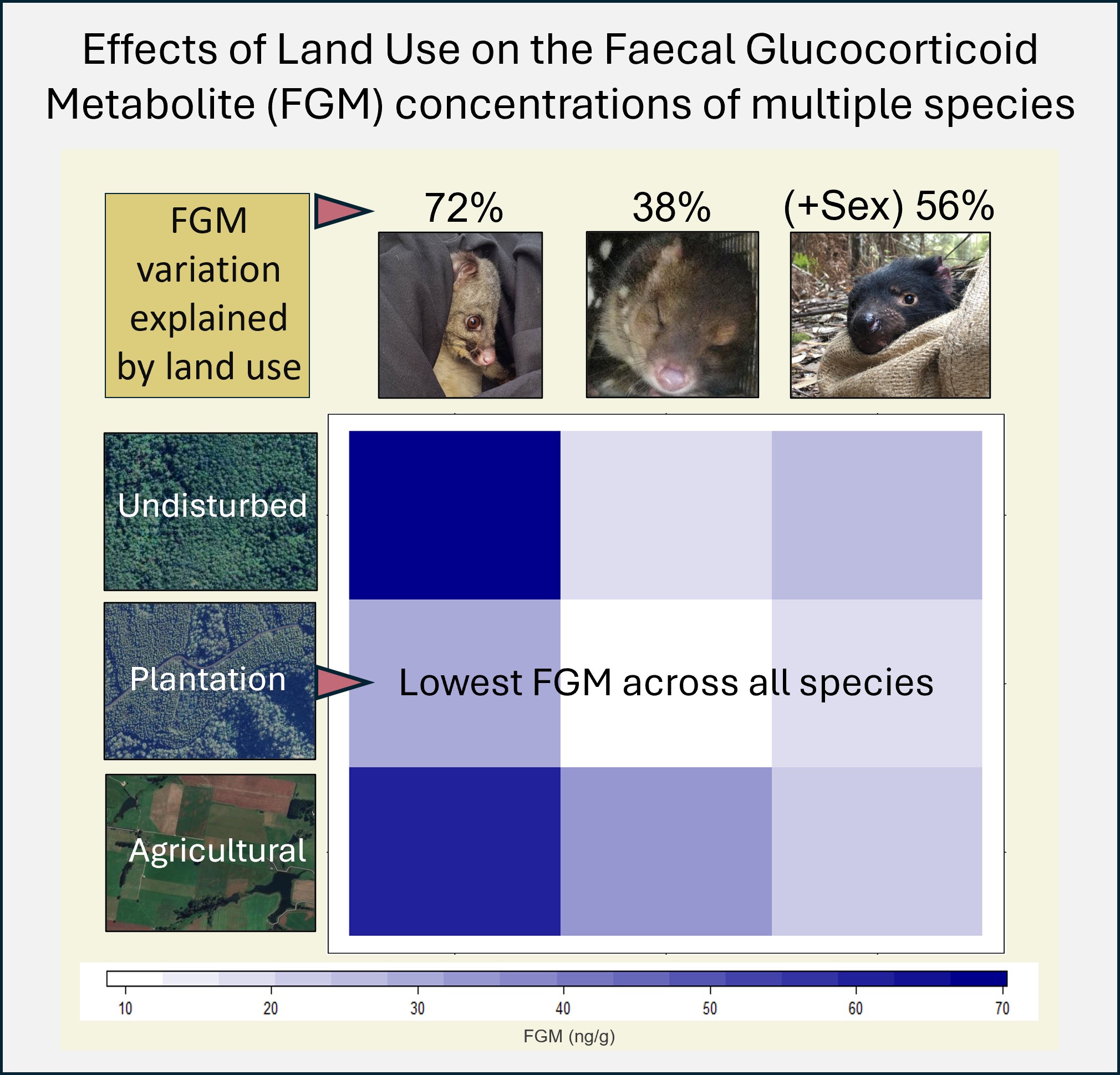

Supplement: graphAbstract2_coae091 [file graphabstract2_coae091.jpeg]
